# “They heard our voice!” patient engagement councils in community-based primary care practices: a participatory action research pilot study

**DOI:** 10.1186/s40900-020-00232-3

**Published:** 2020-09-21

**Authors:** Julie Haesebaert, Isabelle Samson, Hélène Lee-Gosselin, Sabrina Guay-Bélanger, Jean-François Proteau, Guy Drouin, Chantal Guimont, Luc Vigneault, Annie Poirier, Priscille-Nice Sanon, Geneviève Roch, Marie-Ève Poitras, Annie LeBlanc, France Légaré

**Affiliations:** 1 VITAM – Centre de recherche en santé durable, CIUSSS de la Capitale-Nationale, Pavillon Landry-Poulin, Room A-4574, 2525, chemin de la Canardière, Quebec City, Quebec G1J 0A4 Canada; 2 Tier 1 Canada Research Chair in Shared Decision Making and Knowledge Translation, Université Laval, Quebec City, Quebec Canada; 3 Department of Family Medicine and Emergency Medicine, Faculty of Medicine, Université Laval, Quebec City, Quebec Canada; 4 Department of Management, Faculty of Business Administration, Université Laval, Quebec City, Quebec Canada; 5 Patient partner, Quebec City, Quebec Canada; 6 Clinic Manager, Quebec City, Quebec Canada; 7 Centre Hospitalier Universitaire de Québec – Université Laval Research Center, Hôpital Saint-François d’Assise, Quebec City, Quebec Canada; 8 Faculty of Nursing, Université Laval, Quebec City, Quebec Canada; 9 Department of Family Medicine, Faculty of Medicine and Health Sciences, Université de Sherbrooke, Chicoutimi, Quebec Canada

**Keywords:** Primary care, Quality improvement, Patient and public involvement, Patient-centeredness, Patient advisory council, Participatory action research

## Abstract

**Background:**

Patient engagement could improve the quality of primary care practices. However, we know little about effective patient engagement strategies. We aimed to assess the acceptability and feasibility of embedding advisory councils of clinicians, managers, patients and caregivers to conduct patient-oriented quality improvement projects in primary care practices.

**Methods:**

Using a participatory action research approach, we conducted our study in two non-academic primary care practices in Quebec City (Canada). Patient-experts (patients trained in research) were involved in study design, council recruitment and meeting facilitation. Advisory councils were each to include patients and/or caregivers, clinicians and managers. Over six meetings, councils would identify quality improvement priorities and plan projects accordingly. We assessed acceptability and feasibility of the councils using non-participant observations, audio-recordings and self-administered questionnaires. We used descriptive analyses, triangulated qualitative data and performed inductive thematic analysis.

**Results:**

Between December 2017 and June 2018, two advisory councils were formed, each with 11 patients (36% male, mean age 53.8 years), a nurse and a manager practising as a family physician (25% male, mean age 45 years). The six meetings per practice occurred within the study period with a mean of eight patients per meeting. Councils worked on two projects each: the first council on a new information leaflet about clinic organization and operation, and on communications about local public health programs; the second on methods to further engage patients in the practice, and on improving the appointment scheduling system. Median patient satisfaction was 8/10, and 66.7% perceived councils had an impact on practice operations. They considered involvement of a manager, facilitation by patient-experts, and the fostering of mutual respect as key to this impact. Clinicians and managers liked having patients as facilitators and the respect among members. Limiting factors were difficulty focusing on a single feasible project and time constraints. Managers in both practices were committed to pursuing the councils post-study.

**Conclusion:**

Our results indicated that embedding advisory councils of clinicians, managers, patients and caregivers to conduct patient-oriented quality improvement projects in primary care practices is both acceptable and feasible. Future research should assess its transferability to other clinical contexts.

## Plain English summary

Patients and their caregivers would be better served if patients themselves were involved in advising primary care clinics and coming up with new projects to improve services. We tested a new kind of patient advisory council for primary care clinics to see how well it worked. Council members would be patients, caregivers, clinicians and managers. We set up these councils in two primary care clinics in Quebec, Canada, and they met six times over a year. Meetings were led by patient-experts trained in research. Councils came up with ideas for projects and then chose which ones to work on. Researchers observed the meetings, recorded them and wrote notes. At the beginning and end of the period, council members filled in questionnaires. Their projects were: a new patient information leaflet on how the clinic was organized and how it worked, informing people about existing local public health prevention programs, other ways to involve patients, and improving the appointment scheduling system. Members liked the experience of being on the council. Clinicians and managers liked having patients as facilitators; liked the feeling of respect among members; and the presence of managers gave all members the sense they could really change things. But it was hard to come to a decision about which projects could be achieved in the real world; people felt they didn’t have enough time; and they were concerned that the voices of a bigger variety of patients should be heard. It’s important to involve patients in improving primary care. Our councils could be a good beginning.

## Background

Patient experience is now recognized as one of the three key components to achieving high quality healthcare organization [1]. Engaging end-users is expected to promote the development of patient-oriented quality improvement initiatives with direct impact on the functioning of health services and patient health outcomes [2, 3]. However, health professionals need support to fully integrate patients’ perspectives in quality improvement actions and use experiential data from a diversity of patients [4]. As the first-line and principal healthcare providers for the population, community-based primary care practices (CBPCPs) must engage in the transformation of the healthcare system through patient partnership [5–7]. Primary care organizations and health authorities worldwide are encouraging patient engagement in CBPCPs to foster a culture of patient partnership in all dimensions of care, including direct care provision but also in organization of services [6–9]. Patient engagement can be defined as active partnership among all stakeholders, including patients and their relatives, as well as professionals, working together to improve healthcare delivery [10]. Moreover, patient engagement methods that align with the particularities of the CBPCP setting have yet to be developed. Indeed, CBPCPs have little incentive to develop patient-oriented quality improvement projects and have no dedicated staff or funding for this purpose.

Patient engagement can occur at a variety of different levels (or “intensity”). High-level engagement could consist of patients engaging in the governing boards of clinics or patient advisory councils (PACs), while low-level engagement could consist of a suggestion box in a clinic, or participating in surveys [11, 12]. Evidence regarding the modalities and impact of high-level engagement strategies, especially in primary care, is limited [13–18]. This may explain why few practices succeed in implementing these strategies [13, 19, 20] or succeed in sustaining the involvement of patients in a meaningful way over the long term [15, 21, 22]. However, effective strategies for high-level engagement for CBPCP has not yet been identified.

Thus, we sought to assess the acceptability and feasibility of embedding advisory councils of clinicians, managers, patients and caregivers to conduct patient-oriented quality improvement projects in primary care practices.

## Methods

### Study design

We used a participatory action research [4] approach following the iterative cycles of *Reflect*, *Plan*, *Act* and *Observe* [23, 24]. Details of our development methods and participatory approach can be found elsewhere [25]. Briefly, the study was conducted in partnership with four patients trained in the science of patient partnership in research [26–28], referred to as ‘patient-experts’. A study coordinator (JH) was in charge of study planning and supporting the patient-experts and all other practical aspects of organization [25]. Patient-experts were in charge of recruiting council members and facilitating council meetings. They were also involved in the design of meeting content and tools. A steering committee included three health and social sciences researchers, two family physicians, two nurses, a coordinator from the Quebec Practice-Based Research Network (QPBRN), and one of the patient-experts (JFP).

### The EQUIPPS model

We refer to the new advisory councils as EQUIPPS, standing for *ÉQUIpes Patients, Proches aidants, Soignants* (in English, “patient, caregiver and healthcare provider teams”) [25]. The first version of the EQUIPPS model was co-designed by the steering committee guided by a review of the literature and guidelines [19–21, 29–32]. The councils aimed to create partnership at the organizational level of the CBPCP according to the patient engagement frameworks underpinning the model [33–37].

### Study setting

In the province of Quebec (Canada), most CBPCPs are accredited family medicine groups (FMGs) [38]. This study took place in two privately-owned accredited FMGs in Quebec City (QC, Canada), “CBPCP-A” and “CBPCP-B”. Characteristics of the two CBPCPs can be found in the published protocol [25]. No other research or patient involvement activity had taken place in these CBPCPs before.

### Participants

Based on available literature on conducting PAC, each council was to include 12 patients and/or caregivers, one clinician and one CBPCP manager [19–21, 29, 30]. For pragmatic reasons, inclusion criteria were broad: willingness to participate and availability during the study period. In addition, CBPCP managers (with a medical or administrative background) and clinicians (family physicians or primary care nurses) had to be practising at the participating CBPCP as their main activity. Managers and clinicians were recruited during a kick-off meeting in their CBPCP. Patients and relatives had to be registered at the CBPCP, 18 years old or over, able to maintain a certain critical appraisal about their own condition or illness and demonstrate interest in improving wellness in the whole community. The main exclusion criteria for patients and relatives were being under the care of the participating clinician, being in an acute phase of their disease, having had a conflict with the clinic or behaved inappropriately with clinic staff, and not being fluent in French. No restrictions applied regarding clinical condition or illness. The recruitment of council members followed three steps as recommended by Université de Montréal (Direction Collaboration et Partenariat Patient) guidelines [39]: 1) identification by health professionals working at the CBPCP or directly through a call for participants in the waiting room, 2) phone interview with a patient-expert, 3) face-to-face interview with two patient-experts. Patient experts were recruited through the patient experts network of the Quebec Practice-Based Research Network (QPBRN) [40]. Further details of the council member recruitment process can be found in the published protocol [25] .

### Proposed advisory councils

For this study, we planned to schedule for each CBPCP council six 90-min meetings every 6 weeks, over a 12-month period, facilitated by a mixed-sex pair of patient-experts (PNS and LV in CBPCP-A and AP and JFP in CBPCP-B). We defined the attendance threshold for holding the meeting as presence of the manager, the clinician and a minimum of six patients and/or caregivers. Facilitators had to ensure that the program was followed, that the meeting proceeded in a friendly and collaborative atmosphere, that all council members had their chance to speak, and that any controlling behavior or disruption was avoided.

The first meeting, as proposed in the model, consisted of training for council participants on healthcare system organization and patient engagement. This training was co-designed with the four patient-experts who assessed and revised the comprehensibility of all concepts and terms commonly found in health system institutions, practice organization, as well in health systems research settings. Thus all the terms, definitions and explanations provided were fully comprehensible to the patient-experts [41]. After this first meeting, the meetings proceeded as follows: Meeting 2: brainstorming to identify patient-oriented quality improvement priorities; Meeting 3: thematization and prioritization of ideas according to their relevance, usefulness and feasibility as inspired by the Child Health and Nutrition Research Initiative criteria [42]. These criteria for priority setting are widely used in numerous settings [43] and could be adapted to fit well with the timelines, with the requirement of comprehensibility for a broad spectrum of education levels, and other requirements of our project. After prioritization, councils members worked during Meetings 4 and 5 on potential initiatives and discussed actions to address the selected priorities. The Meeting 6 consisted in a focus group on members’ experience of being on the council, motivation for being on the council and expectations of it, perceptions of patient involvement, barriers and facilitators to patient involvement in the councils, and perception of the council’s impact on the CBPCP during the study period.

Facilitation, activities proposed for the meetings and resources were adapted to the needs of the council members from one meeting to the next.

### Outcomes criteria

The feasibility of implementing our council model in the participating CBPCPs was to be assessed based on four dimensions: process, resource, management and scientific [44]. For each dimension, we defined criteria and thresholds (Table 1). We also assessed the acceptability of the advisory council model based on satisfaction of participants at the end of the study rated on a 0 to 10 scale, perceived impact of the council (yes/no/not sure) and qualitatively on perceptions of council members about their experience of involvement in the councils, the barriers and facilitators to their involvement, their interactions and the power relationships among council members.

**Table 1:** Feasibility criteria and pre-defined thresholds for determining feasibility

Feasibility dimension	Criteria	Threshold
Process issues	Retention of CBPCP and council members throughout the study	The 2 CBPCPs are still involved in the study at the end of the 12-month process
	Number of meetings planned and held during the 12-month study period	At least four out of the six planned meetings take place
	Attendance of council members	At least six patients, the clinic manager and the clinician attend each meeting
Resource issues	Time required to recruit council members and organize meetings	Total time to complete recruitment of council members is under 6 months
	Communication between research team and council members	The CBPCPs are able to respond to the study coordinator’s requests and organize meetings
	Resources needed to organize and hold all meetings	Resources needed to organize meetings and compensate council members are covered by the study funding
Management issues	Acceptability of meeting format according to study personnel, clinics and council members	The research team receives no complaints from clinics, patient-experts or council members about the functioning and agenda of the meetings
	Interactions among council members during the meetings	Patient-experts do not encounter difficulties in facilitating the meetings.
	Capacity to overcome challenges	The project overcomes the challenges and proceeds as planned
Scientific issues	QI and patient-oriented research topics that are identified during the meetings	Each council comes to a consensus on at least one QI topic or patient-oriented research question to be addressed
	Projects and actions that are shaped around these topics	Proposals are made by council members to respond to identified priorities and to improve services or conduct research projects

*Abbreviations*: *CBPCP* community-based primary care practice, *QI* quality improvement

### Data collection

Data were collected from all council members using structured questionnaires distributed at the first and sixth council meetings, and from non-participant observers (JH and SGB) using a structured log-book and from audio-recordings of the meetings. Development of all study materials (questionnaires, data collection grids) were guided by existing literature [31, 32, 45] and validated by the steering committee and patient-experts. Details on the materials are available in the protocol [25]. Briefly, at the first meeting, council members completed a socio-demographic questionnaire and a questionnaire on their knowledge about patient-oriented research, and their motivation for and perceptions about engaging in the advisory council. This same questionnaire was completed by council members at the sixth meeting, with the addition of questions on their perceptions about the impact of the council, satisfaction regarding their participation in the council, as well as barriers and facilitators to sustaining their involvement over the long term. Questionnaires included open-ended questions, multiple-choice questions and statement questions with answers on ordinal 0–10 scales or Likert scales.

### Analysis

We considered a priori that we could conclude the model was feasible if we reached the predefined thresholds in each feasibility dimension (Table 1).

We described quantitative data using frequencies and proportions, or means and standard deviations or median and interquartile range as appropriate. We described data from the initial and final questionnaires on perceptions of council members and in each CBPCP, using means and standard deviation or median and minimum/maximum values for quantitative data, and frequencies and proportions for qualitative data. Analysis was conducted using the SAS 9.4 software (2013, SAS Institute Inc., Cary, NC, USA).

For qualitative analysis, we triangulated methods, data collection tools, data sources and observers. We conducted an inductive thematic analysis of qualitative data collected in the log books, verbatim transcripts of audio recordings of meetings, materials used during the meetings and responses to open-ended questions to analyze council members’ perceptions, barriers and facilitators to patient involvement in the councils. Analysis was conducted during the course of the study by JH using the N-Vivo software (QSR International, NVivo Qualitative Data Analysis Software) [46]. Inferences from the qualitative analysis were combined with results from the quantitative analysis of questionnaires. Quotes are attributed using acronyms that indicate participants’ sex (M for men, W for women), status (P for patient, M for manager, C for clinician) and CBPCP (A or B).

## Results

### Recruitment process and council member characteristics

**Fig. 1:**
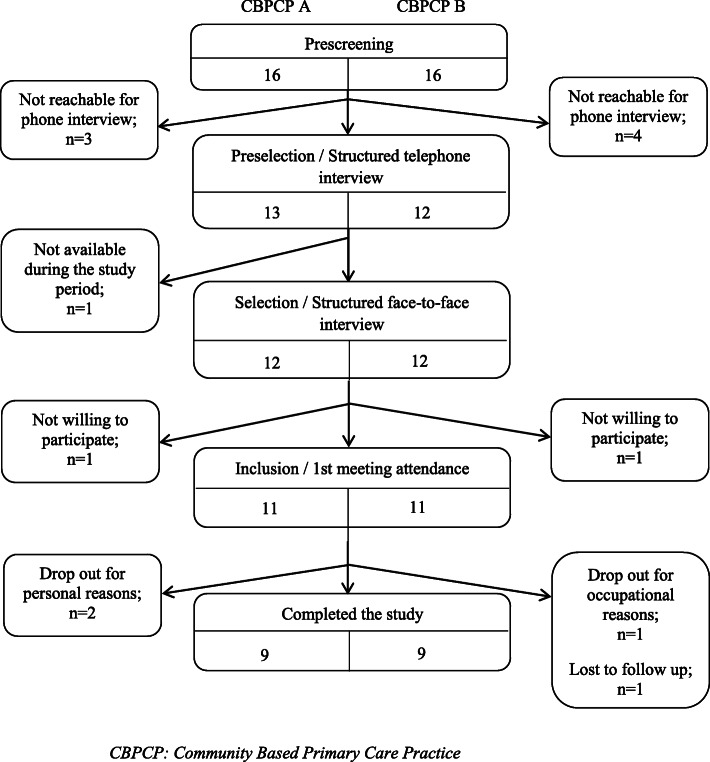
Flow chart of recruitment of patients in the 2 CBPCPs

In CBPCP-A, three patients directly contacted the study coordinator after seeing a call for participants in the waiting room; all other patients were identified by the co-principal investigator of the study (IS), practising there as family physician, and a clinician nurse. In CBPCP-B, three patients were directly recruited by family physicians and the others by a clinician nurse. After recruitment ended (Fig. 1), 11 patients in each council attended the first meeting and signed consent forms. While some of the patients were also caregivers, none of the participants were caregivers only. Patient characteristics are presented in Table 2, and in Additional file 1. In both CBPCPs, the managers were family physicians practising in the clinic (male in CBPCP-A, female in CBPCP-B) and the clinicians were both female clinician nurses. The manager of CBPCP-B had previous clinical research experience.

**Table 2:** Characteristics of patient members of councils

	CBPCP-A (***n*** = 11)	CBPCP-B (***n*** = 11)
Sex, n (%)		
Male	7 (63.6)	1 (9.1)
Female	4 (36.4)	10 (90.9)
Age (years old), mean ± SD (min-max)	58.2 ± 13.6 (32–72)	49.5 ± 16.3 (27–83)
Employment status, n (%)		
Retired	5 (45.5)	2 (18.2)
Employed	3 (27.3)	8 (72.7)
At home / job seeker	3 (27.3)	1 (9.1)
Works in health system, n (%)	3 (27.3)	5 (45.5)
Educational level, n (%)		
Primary/Secondary	2 (18.2)	3 (27.3)
College	3 (27.3)	4 (36.4)
University	6 (54.5)	4 (36.4)

*CBPCP* community based primary care practice, *SD* standard deviation

Members admitted that their family physician’s suggesting it was a main motive for their participation, but that physicians being responsible for determining which patients were eligible skewed recruitment towards specific patient profiles. Both professionals and patients questioned whether council members were representative of the general CBPCP patient population: “This committee must be diversified … you need young people, older people, you need men and women, people … who have different health issues.” (MMA), “We should have people with lower literacy, who are from more vulnerable backgrounds participating because they are [more] representative of the population and we need to hear from them.” (WPB). However, participants were conscious that not everyone wants to participate in such meetings and it may be unrealistic to expect wider representation: “In reality … we take the people who are there and then have to find another way to get the points of view of people who are not necessarily represented on the committee.” (WPB). Council members suggested adding other patient engagement activities to the CBPCP or developing tools for the council to know more about other patients’ perspectives: “We suggested collecting feedback [from CBPCP patients], maybe through a suggestion box or simply by an electronic device for patients to leave their opinion before they leave the CBPCP” (WPB). “It would be useful for the committee to have the main characteristics of the CBPCP patients … there must be data about them … to better know who we are representing” (WPB). To improve diversity of members and maintain motivation, members of both CBPCP suggested regularly renewing members while keeping them involved for a long time: “I would like it to be … a two-year mandate, for example, then every year, half leaves and then they bring new people in.” (MMA). Renewing members on a regular basis would bring new ideas in and mix new and old members, enabling old members to train new members.

### Participants’ expectations and motives for engagement

Expectations of patients and professionals when joining the council were mainly to improve patient services at the CBPCP (Table 3).

**Table 3:** Council members’ perceptions about their experience of involvement in the councils

**Patients’ Perceptions (*N*** **= 22)**	
**Patients’ confidence in their ability to identify CBPCP priorities to improve services and QI**^**a**^**, median (min-max)**	
At the beginning of the study (*N* = 22)	5 (1–10)
At the end of the study (*N* = 15)	8 (4–9)
**Three main motives of patients to engage in the council at the beginning, n (%)**	
To help staff to improve healthcare and services	22 (100.0)
To improve patient experience of care in the CBPCP	20 (78.0)
To improve relevance of research projects	17 (54.0)
**Patients satisfied with the training (in first meeting) (*N*** **= 21)**, n (%)	
Definitely satisfied	21 (100.0)
**Patients perceiving training (in first meeting) as useful for their participation (N = 21)**, n (%)	
Totally agree	20 (95.0)
Not sure	1 (5.0)
**Barriers to participation for patients, n (%)**	
Time constraints	14 (60.0)
Involvement in work or family activities	11 (50.0)
No perceived impact on services to CPBCP patients	11 (50.0)
No perceived impact on patient experience in the CPBCP	11 (50.0)
No funding / financial compensation	1 (4.5)
**Patients’ perception that councils had an impact on the CBPCP at the end of the project (*N*** **= 15), n (%)**	
Yes	10 (66.7)
No	0 (0.0)
Don’t know	5 (33.3)
**Patients’ willingness to participate again in such a council (*N*** **= 15), n (%)**	
Yes	12 (80.0)
No	2 (13.3)
Not sure	1 (6.7)
**Patients satisfaction**^**a**^ **regarding their participation in the council (*N*** **= 15), median (min-max)**	
Overall satisfaction	8 (6–10)
Interactions with other patients	8 (5–10)
Interactions with the clinician	8 (8–10)
Interactions with the manager	9 (7–10)
Interactions with the patient-expert facilitators	9 (8–10)
**Clinicians and managers’ perceptions (*N*** **= 4)**	
**Three main motives of clinicians and managers to engage in the councils, n (%)**	
To improve patient experience of care in the CBPCP	4 (100.0)
To improve practices of CBPCP health professionals	4 (100.0)
To contribute to developing new scientific knowledge	4 (100.0)
**Barriers to participation for clinicians and managers, n (%)**	
Time constraints	4 (100.0)
No perceived impact on services to CPBCP patients	1 (25.0)
Lack of confidence in researchers	2 (50.0)
No funding / financial compensation	0 (0.0)

*POR* Patient Oriented Research, *QI* Quality Improvement, *M* Men, *W* Women, *P* patient, *CBPCP* Community-based primary care practice, *A* CBPCP-A, *B* CBPCP-B

^a^On a 0 to 10 scale, highest values meaning high perception

The objective of the council as perceived by members at the beginning of the study was to co-design new services and improve service quality in the CBPCP. For example, one patient perceived the objective as “to talk about lived experiences of participants” (WPB), “to improve patient experience, services and care, and to collect ideas” (WPB), “to together find services and care practices that could help patients of the CBPCP” (WPB), while a professional perceived the objective as “to improve practices in the CBPCP and respond more specifically to patients’ needs” (WCA). Motivations of professionals were patient experience improvement, health professionals’ practices improvement and developing new scientific knowledge (Table 3).

### Feasibility

(see Feasibility thresholds, Table 1)

#### Process (eg. meetings, work topics, member mandates, council relationship with CBPCP)

In each CBPCP, the six meetings occurred over a 12-month period, with a mean of 2 months between each meeting in CBPCP-A and 6 weeks in CBPCP-B. The preferred frequency of meetings according to members was one per month. Clinician, managers and patient-expert facilitators attended all the meetings. A median of eight patients were present at each meeting (between seven and ten) (see Additional file 1). Four patients dropped out of the study, two in each CBPCP; one after the first meeting for occupational reasons, two for time/family constraints, and the last one did not give a reason.

All meetings took 90 min and followed the planned program. Members liked the structured activities suggested in the predefined meeting program during successive meetings and the step-by-step approach to helping them focus on work topics. “There were a lot of [possible] work topics and ... having to focus, as a research team, I found that interesting. There are other things I would have liked to work on, but they can be addressed later, because we had to make choices.” (MPA). They also enjoyed the flexibility of the approach and the fact that they could choose the direction of the council’s work: “We didn’t necessarily limit ourselves to the [what was suggested in the] program … we proceeded based on the here and now.” (WPB). All members were satisfied with the training provided by the patient-experts during the first meeting and 95% declared it was useful for participation in the council (Table 3). Members judged it necessary but felt that too much information was delivered all at once during the first meeting, so that participants could not link the information with their subsequent work. Members suggested splitting the training into several phases and training council members on topics that matched their needs and proposals as they arose.

In both CBPCPs, members suggested expanding the mandate of the council to being a resource for all patients of the CBPCP through activities such as producing a newsletter or a journal or holding events with the aim of informing people about the council, raising interest in potential new members, and promoting and enhancing the councils’ activities. CBPCP-B proposed giving the council its own identity with a name, an office, and an email address. People were concerned about the credibility of the council in the organizational structure of the CBPCP at the end of the research process: “From the outside, people ask what kind of committee are you? and then they say, Well, that’s a fake committee.” (MPA). Whether the councils should be autonomous or mandated by the board of directors of the clinic was discussed, with members emphasizing that they should be able to continue to make proposals based on their own priorities rather than be mandated by the CBPCP to reflect on pre-specified topics. The idea that councils could have a representative who attended certain meetings of the board of directors of the clinic came up in both CBPCPs. In this case, the positioning and the functioning of the councils would have to be clarified and validated by the board.

#### Resources (remuneration for recruitment and coordination time, expenses)

Time needed to complete recruitment process was less than 6 months in both CBPCPs (3 months in CBPCP-A and 5 months in CBPCP-B).

Financial resources needed to conduct the study were covered by study funding. They related to person-funding: funding for patient-experts, patient-participant expenses (CAD$60 per meeting and travel fees), and study coordinator funding. Professionals attending the council (manager and clinician) were made available by their CBPCP without compensation. Other expenses were renting the meeting rooms from the CBPCP stockholders’ group, accommodation costs (food/drinks) and stationery costs. Materials needed for the councils’ activities were printing, stationery and a video projector. Participants believed that remuneration would not limit participation (Table 3): “There would be just as many people without financial compensation” (WPB). On the other hand, participation was seen as time-consuming and in competition with other activities such as work, family or leisure for both patients and professionals (Table 3): “You might need to stop your other activities” (WPB).

#### Management (eg. member interaction, reporting, leadership, meeting atmosphere, participation, credibility, facilitation)

No complaints from patients, clinicians, managers, and patient-experts were registered during the study. Between the meetings, council members interacted only with the study coordinator and not with each other. After each meeting, the study coordinator sent a report by email to the council members with a reminder of the date and agenda of the next meeting. The study coordinator, the facilitators and the steering committee also searched for practical information and relevant scientific literature to support the ongoing work of the council members and presented it to council at the following meeting. Examples are a list of locally available prevention programs and a scientific review of effective ways to collect patient experiences. No additional work had to be done by council members between meetings. Members agreed that after the end of the study period someone else would have to replace the study coordinator, who was in charge of organization and logistics: “It takes someone and I’m not sure that it could be just a volunteer” (WPB).

All members contributed to meetings by giving their point of view. An opportunity for each member to speak and a friendly atmosphere were established through the facilitation by the patient-experts, who reported that they had no difficulty facilitating the meetings. Patients were highly satisfied by the quality of the interaction among participants (Table 3). Council members identified that behavioral skills were needed for participation such as respect, communication, being open-minded and self-confident and being able to question one’s opinions “We don’t all agree, but it’s still about respecting others even if we don’t agree.” (WPB). However, specific “technical” skills were not needed to participate: “I feel like we all were able and willing to participate. It was a patients’ meeting so we didn’t expect patients to think like doctors or vice versa” (MPA), “We all had our own particular strengths and putting them all together really helped us to make better progress” (WPB). However, we noticed that in each council, the clinician, who was a female nurse practitioner, was addressed less often than the managers (who were also family physicians), and that in each council one patient was more reserved and isolated than the others (the youngest member in CBPCP-A, and the oldest and only male member in CPBCP-B). Two patient members declared that they didn’t feel sufficiently self-confident or valued for their participation: “I feel like I am not the right person to pursue this” (MPA). Managers also pointed out that some patients contributed mostly with their own personal experience, while others took a more objective perspective: “Patients generally rely on their personal or individual experience” (WMB); “In a committee like this you can’t go with your own feelings. You have to go with something higher because you represent something more than your own lived experience or your own feelings about a situation” (WMB), “You need to have a global vision, not a personal vision of care … it shouldn’t be a place where we discuss our own problems, our own difficulties. If it’s a difficulty that everyone experiences, then yes.” (WMB).

The manager was the council member the most frequently addressed by the other participants at each meeting. Managers were seen as the ones who knew and who could confirm information. Patients were very satisfied with the interactions with the manager (Table 3), they stated that the presence of the manager was a key reason for the success of the councils: “Participation of the manager is a prerequisite” (WPB), “The manager is here so he / she heard our voice” (WPB). “[The presence of the manager or representative] is very important otherwise there would be a high risk of demotivation if there’s a feeling that the council can’t change anything” (WPB). Some ideas discussed by patients at meetings had already been implemented by the managers before the next meeting, increasing the feeling of efficacy of the council members. For instance, documents were made available to patients in CBPCP-A, and work began on a car park in CBPCP-B to facilitate access for people with disabilities. The presence of clinicians was also appreciated by patients, but they mainly addressed their questions or complaints to the manager. Professionals were concerned to explain and help the council work on topics that were feasible and relevant: “My fear was when I really saw that we were moving towards [discussing] things that we just couldn’t change. So if [the professionals] weren’t there to say, ‘We won’t put any energy into this, it’s not working, or it’s already happening’, there would be so much time wasted” (WPB). Explanations given by the professionals in response to criticisms made by patients reduced the mistrust of patients in the health system “Frustration is immediately removed when you understand the reason behind it” (WPB).

Council members liked the facilitation by the patient-experts, who were not patients of the CBPCPs (Table 3): “I liked it that we were led by [neutral] facilitators and then by researchers, it made it easier … if we continue with a council of patients, it will take someone neutral to manage that too.” (MMA).

#### Scientific (ideas, priorities, outputs, achievements)

After the brainstorming sessions, 36 ideas were raised in CBPCP-A and 50 in CBPCP-B. The ideas were then grouped into six themes by the study coordinator. The classification was revised and validated by council members. Themes were then prioritized based on feasibility, utility and impact. Based on the results of the prioritization, each CBPCP chose two out of the six themes to focus on, and then split into two subgroups (Groups 1 and 2) to focus on their two themes. One professional participated in each subgroup (either the nurse or the manager).

At the end of the process, the subgroups’ work delivered four outputs as follows:

- CBPCP-A, Group 1: A revised information leaflet for new patients. Adapted to the literacy level and needs of patients, the leaflet presented how the CBPCP functions (services provided, opening hours) and explained the decision process when facing a health issue, depending on the emergency of the situation and on the availability of services. A dissemination process was also discussed that would ensure patients get the leaflet when needed. Since the end of the sixth meeting, the leaflet had been made available to all patients and is being used in the practice.
- CBPCP-A, Group 2: A program of communication to better disseminate information about existing health prevention programs in the province of Quebec. They planned to use existing materials or develop new ones on these programs, and to broadcast them on TV screens in the clinic waiting rooms. They also proposed integrating the programs into the health awareness calendar (e.g. mental health week) published by the health authorities.
- CBPCP-B, Group 1: Proposals for the consultation scheduling system (opening hours, call center, internet platform) to improve the process and reduce wait times. The group proposed organizing the automated phone menu differently and made structured proposals for new services that would fit the needs of patients (e.g. telemedicine and online visits, short visits and suggested alternative time slots).
- CBPCP-B, Group 2: Recommendation to improve the patient experience in the clinic and make the clinic more patient-centered. The group proposed several measures such as further training of professionals (e.g.: in patient partnership, alternative medicine, vulnerable population profiles), new activities involving patients and professionals (conferences, sports groups, a journal, events) and implementation of methods to gather the experience and needs of all patients (suggestion box, patient experience questionnaire).
  The proposals made by CBPCPA group 1 and by the two groups in CBPCPB have been transmitted to the governing board of the CBPCP for further action.

### Members’ perceptions of their participation in the councils

On a scale of 1–10, participation in the councils increased patients’ confidence in their ability to identify CPBCP priorities to improve services and quality of care from 5/10 to 8/10. On a scale of 1–10, patients were satisfied overall (8/10) with their participation in the councils and 80% would participate again (Table 3). Indeed, participation in the councils was a very positive experience for all members (“I adored my participation” WPB). Patients emphasized the positive effect of the group: “It was a wonderful experience, the diversity of members in terms of age is really positive for the clinic and for what we’re doing … we make better decisions and suggestions [together], because it’s difficult when you’re alone to see with the eyes of the majority.” (WPA). “It is great to see the magic of the group, the group effect is incredible” (WPB). However, two members declared that it was difficult to feel comfortable in the group, which they considered too large; they felt more at ease in the small group discussions. Other members emphasized that working in small groups was more productive.

As a direct benefit of their participation, patients gained a better understanding of the functioning of the clinic and its constraints: “It is the sense of belonging. Because now I don’t see my clinic the same way.” (WPB). Being on the councils changed their opinion of the CBPCP and of the health system: “You say to yourself, ‘Well, if they don’t answer my calls, we’ll find a solution.’ Instead of saying ‘It’s me AGAINST them’, it’s me AND them, [and] if the health network doesn’t meet my needs, it becomes like my problem too’” (WPB). “There is a popular saying: ‘It’s by talking that we come to understand each other’. The suggestions we make help us … to become aware that contact with CBPCP staff can be more human, and then it becomes … not just a team effort, but almost a family atmosphere.” (WPB).

### Members’ perceptions of impact of councils on CBPCPs

Two-thirds of patient participants felt that the council had had an impact and one-third weren’t sure (Table 3). Although patient members directly perceived some impacts on the practices thanks to the manager implementing certain ideas from one meeting to the next, participants thought the study period was too short to measure any real and deep impact. “It was one meeting per month and six meetings, so of course we couldn’t get into big projects”. However, this did not discourage them from planning to pursue their ideas: “… but if we decided to go ahead with a patient committee, then we could work on things that are longer-term and … require a little more investment too.” (MPA). Two members felt that the objectives of the councils were not sufficiently clear at the beginning: “At first it was a bit of a mystery to us” (WPA), and that the impact of the council was not as great as they had hoped: “[I kept asking myself] are we on the right track or are we slipping, or is it realistic, concrete, doable? … I had a question about that. Not because I didn’t find it interesting, not at all, but about the conclusions.” (WPB).

For professionals, participation in the council changed their views on patients’ understanding of the functioning of the CBPCP, of the health system, and of the literacy levels of their patients: “I was not aware of the literacy issue” (MMA), “It makes me realize what patients really face when they try to understand the system” (MMA). Managers both stated that it helped them to identify topics that are relevant for patients: “Sometimes we have ideas that we think are excellent for patients, then.... the committee helped me to see that sometimes we’re completely off track” (MMA). Managers stated that they would pursue the councils after the end of the research project. However, they said that the impact of the councils would depend on the feasibility of ideas and proposals made by patients. Some ideas were too expensive or impracticable. “They do not necessarily realize the costs that their ideas would entail” (WMB). Moreover when patients roundly criticized an aspect of the clinic’s functioning (e.g. private billing), the resulting debate tended to hijack any constructive discussion.

## Discussion

We found that embedding advisory councils of clinicians, managers, patients and caregivers to conduct patient-oriented quality improvement projects in primary care practices was both acceptable and feasible. Our proposed advisory council mostly corresponded to the expectations of participating patients and professionals. In each primary care practice, the council produced pragmatic proposals that could foster patient-centered approaches and improve patient experiences. Participants were highly satisfied with the model. The majority perceived the council’s impact on the clinic and wanted to pursue the council after study completion. The main motivation for both professionals and patients to participate in the councils was to improve practices and patient experience. Involvement of a manager, facilitation by patient-experts, and the fostering of mutual respect were identified as key to the council’s impact. Main limiting factors were difficulty focusing on a single feasible project, member profiles not being representative enough over the overall patient population, and time constraints. Overall, these results lead us to make the following observations.

Our model differs from patient advisory councils reported in the literature in two components [4–6]: systematic attendance by a member of the governing board and a member of the healthcare team of the clinic, and facilitation by patient-experts who were not affiliated with the clinic. These two components, both mentioned as strengths of the model by participants, are not found in patient councils reported in the literature [7–9]. The presence of professionals from the clinics proved their commitment to supporting the councils and testified to the importance they attached to it. It created a direct link between council members and clinic governance. This was seen by participants as key to their proposals being taken seriously and put into practice. They stressed that the councils must have status and credibility in the clinic governance structure to ensure recommendations were followed. Moreover, councils advocated for even greater involvement of patients in practice governance, suggesting that a patient representative always attend governing board meetings. While clinician presence was important, it was the patient-expert facilitators who rapidly put the participants at ease, created an atmosphere of mutual respect, strengthen the links between patients and professionals, dispelled misunderstandings, and refocused discussion when it digressed from the task. Being external to the clinic or “neutral” enabled them to reduce the professional-patient power differential and defuse other such communication problems.

The representativeness of the patients was questioned by participants as it is in the literature [5]. This issue should be carefully taken into account when recruiting patients. Patient engagement strategies must not reinforce health inequities by involving only the best educated and most socially advantaged patients. This risk could be mitigated by using several strategies with a variety of engagement levels [47, 48], e.g. surveys or suggestion boxes (low level), or participation of a patient council member in the governing board (high level). However, beginning patient engagement strategies with highly motivated persons provided the necessary impetus before extending strategies to other participant profiles [49]. Several additional recruitment methods could result in a council that better represents clinic patients. For example, patients on the council could recruit in the waiting room through an information booth. Participants in both councils proposed members be mandated for a short period with regular partial renewal of participants to diversify profiles. One council proposed that councils themselves become responsible for the patient partnership strategy/policy in the clinic. Among other activities, they could make connections with patients who enquire about patient representatives and their projects, giving councils access to a broader range of patient perspectives [50, 51].

Regarding organizational aspects of the councils, our findings are in line with available literature on patient advisory councils [4, 7–9]. Frequency of meetings must balance time required from participants with the desired progress on projects. Time constraints were raised as the first limitation to participation in the council. Logistical and organizational support to prepare meetings and smooth council operations are a necessity. In our study, this role was played by our study coordinator and funded by our research grant, but this role would have to be assumed by another person, at least at the beginning of the council to ensure the council starts off successfully. Participants agreed that this time-consuming task would need to be funded by the healthcare institution to compensate the patient or professional who assumed the role. Based on our experience, the necessary resources to be made available by the clinic for the councils to function optimally are 1/ part-time staff for organization and coordination, 2/ meeting room equipped with videoprojector, 3/ small supplies for each meeting (stationery and snacks), 4/ availability of 2 professionals (clinician and manager) during the meetings.

Another concern was the large scope of the council’s work plan. In our study, participants were supported in a structured step by step approach to define their agenda and some participants found the initial range of possibilities too broad. Setting a clear agenda and presenting it to patients at the beginning of their mandate might reassure them. However, it must not hamper the council’s freedom to propose its own agenda and define its work topics. Receiving regular feedback from the clinic on their activities would also help identify successes and ensure continuing motivation [16].

Our study has some limitations. First, the two participating CBPCPs that volunteered for the study were both high-volume clinics in an urban area and thus not representative of the all CBPCPs in the province of Quebec. Second, the selection of patient council members by CBPCP staff might have biased them towards more highly-educated patients (as seen in CPBCP-B, see Table 2) or those with a more favorable attitude towards the clinic. Representativeness was raised by both councils and members proposed solutions for diversifying patient member profiles. Third, the study period was too short to measure the impact on practices and patients’ experiences at the CBPCP. While the number of meetings planned was sufficient to create group cohesion, identify priorities and plan initiatives, it was too short to implement the initiatives and measure their impact. However, this was a pilot study, and a future study will improve the co-designed model and evaluate a larger and more diversified sample, as proposed by the UK Medical Research Council guide [52]. The strength of our study is its participatory approach, and a co-designed model that is also theory-based [33], responding to the needs of end-users and transferable to other CBPCPs. Moreover our assessment of participants’ experience of the engagement process enabled us to propose an adjusted model, such step-by-step approach remains rare in studies on patient participation [17].

Our results provide a model for engaging patients that is in accordance with the recommendations of the Patient Medical Home model proposed by the College of Family Physicians of Canada (8.6 pillar: Patient participation and formalized feedback mechanisms) [7]. For patient engagement strategies to be realistic in primary care practices, they have to fit seamlessly into standard practices. Through the council, patients must be part of ongoing planning and evaluation. In spite of time constraints, both patients and professionals in our study declared that it was worth it if a true impact was rapidly perceived. Our study also confirmed that making practices more patient-centered benefits the experience, satisfaction and well-being of both patients and professionals [13, 53–55]. Moreover, our participants themselves suggested how the EQUIPPS council model could be improved in the areas of patient member representativeness, management, and legitimacy and motivation. We sought to explore a method for engaging patients in quality improvement of services at the organizational level rather than at the level of how specific diseases are managed. Our model of council lays the foundation for patient engagement at the CBPCP level and could lead toward the development of further councils that could focus on designing and improving services for specific diseases or health issues (eg maternal/obstetrical services).

## Conclusion

Patient engagement activities should be integrated in all primary care clinics. Our results indicated that embedding advisory councils of clinicians, managers, patients and caregivers to conduct patient-oriented quality improvement projects in primary care practices is both acceptable and feasible. They also suggest that primary care professionals are keen to give a voice to patients, and patients are keen to contribute their voice. Future research should assess the transferability of advisory councils to other clinical contexts.

## Supplementary information

**Additional file 1.** Members’ characteristics and attendance of meetings.

**Additional file 2.** GRIPP2 short form checklist.

## Data Availability

The datasets used and/or analyzed during the current study are available from the corresponding author on reasonable request.

## Abbreviations

CBPCP: Community-based primary care practice
PAC: Patient advisory council
EQUIPPS: Équipes Patients, Proches Aidants, Soignants (in English, “patient, caregiver and healthcare provider teams”)
FMG: Family medicine group
