# Supplementary material for: “They heard our voice!” patient engagement councils in community-based primary care practices: a participatory action research pilot study
Source: Res Involv Engagem. 2020 Sep 21;6:54. doi: 10.1186/s40900-020-00232-3 (PMC7507740; doi:10.1186/s40900-020-00232-3)
Supplement: Supplementary file 1 — Additional file 1. Members’ characteristics and attendance of meetings. [file 40900_2020_232_MOESM1_ESM.docx]

Additional file 1: Members’ characteristics and attendance of meetings.

| Council member | Sex | Age | M1 | M2 | M3 | M4 | M5 | M6 |
| --- | --- | --- | --- | --- | --- | --- | --- | --- |
| **CBPCP-A** | | | | | | | | |
| Manager | M | 51 | y | y | y | y | y | y |
| Clinician | W | 34 | y | y | y | y | y | y |
| Patient 1 | M | 46 | y | n | y | y | y | y |
| Patient 2 | M | 32 | y | y | y | y | y | y |
| Patient 3 | M | 63 | y | n | y | y | n | y |
| Patient 4 | W | 64 | y | y | n | y | n | y |
| Patient 5 | W | 68 | y | y | y | y | y | y |
| Patient 6 | M | 56 | n | y | y | n | n | y |
| Patient 7 | M | 72 | y | y | y | y | y | y |
| Patient 8 | W | 38 | y | y | y | y | y | y |
| Patient 9 | M | 64 | y | y | n | n | n | n |
| Patient 10 | M | 71 | y | y | y | y | y | y |
| Patient 11 | W | 66 | y | y | y | y | y | y |
| **CBPCP-B** | | | | | | | | |
| Manager | W | 51 | y | y | y | y | y | y |
| Clinician | W | 44 | y | y | y | y | y | y |
| Patient 1 | W | 65 | y | y | n | y | n | y |
| Patient 2 | M | 83 | y | y | y | y | y | y |
| Patient 3 | W | 60 | y | y | y | y | y | y |
| Patient 4 | W | 40 | y | y | y | y | y | y |
| Patient 5 | W | 46 | y | y | y | y | y | y |
| Patient 6 | W | 49 | y | n | n | n | n | n |
| Patient 7 | W | 34 | n | y | y | y | y | y |
| Patient 8 | W | 37 | y | y | y | y | n | y |
| Patient 9 | W | 27 | y | y | n | n | n | n |
| Patient 10 | W | 43 | y | y | y | n | y | y |
| Patient 11 | W | 61 | y | y | y | y | y | y |

M, Men, W, Women; y, yes; n, no; M1 to M6, Meeting 1 to Meeting 6.
