# Supplementary material for: “They heard our voice!” patient engagement councils in community-based primary care practices: a participatory action research pilot study
Source: Res Involv Engagem. 2020 Sep 21;6:54. doi: 10.1186/s40900-020-00232-3 (PMC7507740; doi:10.1186/s40900-020-00232-3)
Supplement: Supplementary file 2 — Additional file 2. GRIPP2 short form checklist. [file 40900_2020_232_MOESM2_ESM.docx]

| **Section and topic** | **Item** | **Reported on page No** |
| --- | --- | --- |
| 1: Aim | Report the aim of PPI in the study | 6 |
| 2: Methods | Provide a clear description of the methods used for PPI in the study | 7-8-9 |
| 3: Study results | Outcomes—Report the results of PPI in the study, including both positive and negative outcomes | 11-12-13-14-15-16-17-18-19 |
| 4: Discussion and conclusions | Outcomes—Comment on the extent to which PPI influenced the study overall. Describe positive and negative effects | 20-21-22-23 |
| 5: Reflections/critical perspective | Comment critically on the study, reflecting on the things that went well and those that did not, so others can learn from this experience | 20-21-22 |

Additional file 2: GRIPP2 short form checklist.
